# The nexus between poverty and maternal healthcare utilization with a focus on antenatal care visits and choice of place of birth in Somaliland

**DOI:** 10.3389/fpubh.2024.1417883

**Published:** 2024-09-18

**Authors:** Khalid Ahmed Abdi, Jayamohan M. K., Mohammed Adem

**Affiliations:** ^1^Department of Economics, College of Business and Economics, Bahir Dar University, Bahir Dar, Ethiopia; ^2^College of Business and Economics, Jimma University, Jimma, Ethiopia

**Keywords:** poverty, maternal healthcare, antenatal care, wealth quintile, Somaliland

## Abstract

**Introduction:**

Poverty poses a significant barrier to accessing healthcare globally, particularly in relation to antenatal care (ANC) visits and the use of childbirth facilities, both of which are crucial for women’s health and fetal well-being. In Somaliland, only 47% of pregnant women attend healthcare facilities for ANC, with a mere 33% receiving care from skilled birth attendants. Despite this, no previous studies have examined the relationship between poverty and maternal healthcare utilization in Somaliland. This study aims to investigate the effect of poverty on maternal healthcare utilization with focus on ANC visits and the choice of place of birth in Somaliland.

**Method:**

Utilizing data from the 2020 Somaliland Demographic Health Survey, a cross-sectional study design was employed, analyzing a nationally representative sample. The sample size used in this study was 3,183 women of reproductive age. Descriptive analysis, negative binomial regression, and multinomial logistic regression were conducted using Stata version 18.0. Diagnostic tests, including Chi-square − 2log likelihood statistic, Akaike Information Criterion, and Bayesian Information Criterion, were employed to evaluate model fit.

**Results:**

Poverty, as indicated by wealth quintile, was associated with reduced ANC visits (adjusted incidence rate ratio (aIRR) = 0.884, 95% CI: 0.791–0.987) among women in poorer households compared to those in richer households. Women in Togdheer, Sool, and Sanaag regions had lower ANC visit rates compared to Maroodi Jeex region (aIRR = 0.803, 95% CI: 0.687–0.939; aIRR = 0.710, 95% CI: 0.601–0.839; aIRR = 0.654, 95% CI: 0.558–0.768, respectively). Women from poorer households had lower probabilities of opting for public health facilities (adjusted relative risk ratio (aRRR) = 0.457, 95% CI: 0.352–0.593) and private health facilities (aRRR = 0.195, 95% CI: 0.111–0.341) over home births compared to women in richer households. Women in Togdheer, Sool, and Sanaag regions had lower probabilities of choosing public (aRRR range: 0.331–0.175) and private (aRRR range: 0.350–0.084) health facilities for delivery over home births compared to women in Maroodi Jeex region.

**Conclusion:**

Poverty significantly impedes maternal healthcare utilization, contributing to lower ANC attendance and preference for home births over public or private health facility births. Addressing these disparities requires initiatives to eliminate financial barriers, such as user fees, and enhance equitable access through community-based health insurance and improved healthcare infrastructure.

## 1 Introduction

According to the World Health Organization, key reasons for high maternal and neonatal mortalities include failure of women to attend antenatal care (ANC) visits and not giving birth at healthcare facilities with skilled birth attendants (1). ANC visits aim to detect and treat potential health issues, promote healthy behaviors, and prepare for safe delivery (2), while skilled birth deliveries, involve the presence of trained health professionals during childbirth to ensure the well-being of both the mother and child (3). Several factors influence ANC utilization and the choice of place of birth, including education, cultural beliefs, healthcare accessibility, quality of services, and economic status (4, 5). As such, education empowers women with knowledge about the importance of ANC and skilled birth attendance, while cultural beliefs and practices can either encourage or discourage the use of these services (6). Accessibility to healthcare facilities, both in terms of distance and transportation, significantly affects utilization rates. The perceived quality of care, influenced by the availability of trained healthcare professionals and necessary medical supplies, also plays a crucial role in a woman’s decision to seek ANC and choose a healthcare facility for childbirth (7).

In low-income and middle-income countries, millions of people face challenges in obtaining timely and appropriate medical care due to poverty, resulting in delayed diagnoses, increased prevalence of preventable diseases, and heightened mortality rates (8, 9), Economic constraints significantly impact the selection of healthcare providers, influencing not only the affordability of services but also the quality and expertise of healthcare professionals accessible to different socio-economic strata (10). In Somaliland, the demographic health survey shows that 61% of women reported lack of money as a major barrier to accessing healthcare, particularly impacting the utilization of maternal and child health services (11). The utilization rate of antenatal care is estimated at 47%, while only 33% of deliveries take place at healthcare facilities, both notably low (11). Public-private partnerships are encouraged to address the challenge of out-of-pocket expenditure, which impedes healthcare utilization (12). Despite these known barriers, no study has yet demonstrated the association between poverty and the utilization of antenatal care services, including choice of place of birth in Somaliland.

Economic hardship limits a family’s ability to afford healthcare costs, including transportation, consultation fees, and necessary medical interventions (13, 14). Poverty not only restricts access to healthcare but also exacerbates other barriers, such as poor education and inadequate healthcare infrastructure (15). In resource-limited settings like Somaliland, where the majority of the population lives below the poverty line, the financial barrier posed by poverty often outweighs other factors (11). A commonly used measure to assess poverty in health research is the wealth index, which is based on household assets, living conditions, and access to basic services (16). This composite measure allows researchers to classify households into different socio-economic strata known as wealth quintiles, providing an understanding of how economic status influences health outcomes and healthcare utilization. The wealth quintile is particularly useful in settings where income data may be unreliable or difficult to obtain, offering a more stable and comprehensive indicator of economic well-being (16, 17).

Somaliland has significant maternal and child health needs, with high rates of maternal and neonatal mortality (11). Understanding the relationship between poverty and ANC utilization and the choice of place of birth is critical to improving these health outcomes. While substantial research exists on the relationship between poverty and maternal healthcare utilization globally (18, 19), studies specific to Somaliland are lacking. Context-specific research is essential for developing targeted interventions that are culturally and economically appropriate. Findings from this study can provide evidence-based insights to inform policy and healthcare practice in Somaliland. By identifying the specific factors associated with ANC visits and choosing appropriate childbirth facilities, the study aims to support the development of effective public health strategies and resource allocation. Addressing disparities in healthcare utilization caused by poverty can contribute to greater health equity in Somaliland. By focusing on the most vulnerable populations, this study aims to promote inclusive healthcare policies that ensure all women have access to necessary maternal healthcare services. Understanding these dynamics is essential for designing effective interventions to improve maternal health outcomes and promote inclusive and equitable healthcare systems (20). This study aimed to investigate the association between poverty and ANC visits and choice of place of birth in Somaliland.

## 2 Materials and methods

### 2.1 Study setting

Somaliland, an autonomous region in the Horn of Africa, declared independence from Somalia in 1991 but remains unrecognized internationally. Covering approximately 176,120 square kilometers with a population of around 3.5–4 million, its capital is Hargeisa. Somaliland has six regions namely: Maroodi Jeex, Awdal, Togdheer, Sahil, Sool, and Sanaag. The economy relies heavily on livestock exports, remittances, and trade, while facing challenges such as poverty, limited infrastructure, and healthcare access issues (11, 21).

### 2.2 Study design, study period and data source

This secondary analysis utilized a population based, cross sectional study design using the Somaliland Demographic Health Survey (SLDHS) dataset conducted in 2020. The SLDHS is a multi-phase research initiative that examines population health, with a focus on maternal and child health and key global health indicators. The SLDHS uses standardized data collection procedures, sampling, questionnaires, and coding.

### 2.3 Study population

All women of reproductive age in Somaliland, aged 15–49 years, who had given birth within the 2 years preceding the 2020 SLDHS were eligible to participate in the study.

### 2.4 Sample size and sampling design

A total of 3,183 women of reproductive age (15–49 years) were included in this analysis. The sample size calculation was based on the demographic health survey guidelines, which consider various factors such as the prevalence of key health indicators, desired precision, design effect, and non-response rate. A detailed method of the sample size, sampling and household listing used is available in the demographic and health survey manual (22). The SLHDS employed a two-stage stratified cluster sampling strategy to capture a comprehensive view of health indicators across diverse populations. Initially, 2,806 Primary Sampling Units (PSUs) were mapped using satellite imagery and local insights, encompassing urban and rural areas. The first stage involved selecting 35 Enumeration Areas (EAs) per stratum per region using probability proportional to size (PPS), ensuring adequate representation. For nomadic populations, often challenging to reach, data were compiled from nomadic link workers and clan elders, identifying 1,448 temporary nomadic settlements (TNS) across the region.

Household listings within the selected EAs provided demographic data, enabling the identification of households with women of reproductive age. Systematic random sampling was then employed to select households for the survey. In each selected EA, a list of all households was compiled. A random starting point was chosen, and households were selected at regular intervals (every nth household) based on the total number of households and the desired sample size. This method ensured that every household had an equal chance of being included in the sample.

Within each selected household, all women aged 15–49 who had given birth within the 2 years preceding the survey were eligible for inclusion. If more than one eligible woman was present in a household, one woman was randomly selected for participation. This systematic random sampling approach ensured that the sample was representative, and that the data collected was robust and reliable.

### 2.5 Study variables

The study focused on two primary outcome variables: ANC visits and the choice of place of birth. Independent variables included wealth index, residence (urban or rural), education level, region of residence within Somaliland, maternal age categories, total number of children, birth order, internet use, mobile phone ownership, and the availability of healthcare providers (nurse, midwife, clinical officer, doctor). The wealth quintile categorizes participants based on economic status by grouping the five wealth quintiles into two as described in the study by Asif et al. (23). Women in the households belonging to the fourth and fifth quintiles were considered as richer households while women in households belonging to the first three quintiles were considered poorer. The residence variable identified whether participants lived in urban nomadic, or rural areas. Education levels were categorized into none, primary, and secondary or higher, reflecting educational attainment. Regional variations within Somaliland were considered, alongside age groups spanning from 15 to 49 years. The total number of children and birth order provided insights into participants’ reproductive history. Additionally, variables such as internet use and mobile phone ownership were included to understand technology access among participants. The type of health service provider was categorized into nurse, midwife, doctor or clinical officer, reflecting variations in healthcare access and service providers across the region. These variables were selected based on their relevance to maternal healthcare utilization and access within the Somaliland context, aligning with demographic health survey guidelines and previous research in the field (24). Measurement of variables followed standard protocols outlined in the SLHDS 2020 report, ensuring consistency and reliability of data across diverse populations and geographic regions within Somaliland (11).

### 2.6 Data collection method

Trained enumerators, selected for their familiarity with local languages and cultural sensitivities, were responsible for gathering information directly from participants using standardized questionnaires. These enumerators underwent extensive training to ensure uniformity in data collection techniques and adherence to survey protocols. Quality assurance measures were integrated at every stage of data collection. Supervisors and field coordinators provided continuous oversight to monitor enumerator performance and resolve any issues encountered in the field promptly. Additionally, regular meetings and refresher trainings were conducted to reinforce data collection protocols and address any emerging challenges.

The survey employed Computer-Assisted Personal Interviewing (CAPI) using smartphones to record responses during interviews. The smartphones, equipped with Bluetooth technology, enabled remote electronic transfer of completed questionnaires from interviewers to supervisors. Supervisors then transferred the data files to the CSWeb server instances whenever internet connectivity was available. Electronic files were then downloaded from the CSWeb server as csdb files and exported to Statistical Package of Social Sciences (SPSS) version 25 software. The CAPI data collection system, developed specifically for the SLHDS 2020, allowed for real-time synchronization of any revisions to the questionnaire.

To further enhance data quality, rigorous validation checks were implemented during data entry. All collected data exported to SPSS version 25, underwent automated checks to flag inconsistencies, missing values, and outliers for further review and resolution. This process helped minimize errors and ensured the integrity of the dataset. Moreover, the SLHDS 2020 adhered to international standards for demographic and health surveys, incorporating best practices recommended by organizations such as DHS Program and WHO. These standards included ethical considerations, participant confidentiality, and data security measures to safeguard sensitive information (11).

### 2.7 Data management

Data management involved the processes of data entry, cleaning, and storage following the initial data collection. Data were exported from SPSS .sav format to Stata version 18 for further analysis. The cleaning process in Stata focused on identifying and correcting errors, inconsistencies, and missing values to improve dataset accuracy. Post-cleaning, data were securely stored and managed to protect participant confidentiality and comply with ethical standards. Access was restricted to authorized personnel involved in analysis and reporting, ensuring the integrity of the dataset.

### 2.8 Data analysis

#### 2.8.1 Descriptive statistics

Percentages and frequencies were used to summarize women characteristics such as places of birth, residence type, region, access to technology, including internet use and mobile phone ownership, healthcare provider availability, such as nurses, clinical officers, midwives, and doctors, problems with accessing health care such as permission from husbands to seek care, financial accessibility (getting money for treatment), distance to health facilities, and reluctance to go alone, and lastly maternal employment status. Means and standard deviation (SD) were calculated for maternal age, total number of children and birth order.

#### 2.8.2 Model selection

The goal of model selection is to identify the statistical model that most accurately represents the underlying reality based on the available data while minimizing information loss. Several goodness-of-fit tests were used in this analysis, including the Chi-square − 2 log-likelihood statistic, Akaike Information Criterion (AIC), and Bayesian Information Criterion (BIC).

##### 2.8.2.1 Chi-square –2log likelihood statistic

We utilized the -2logL statistic to compare models fitted to the same dataset. This metric is valuable for assessing model fitness in the same dataset, where a higher maximum likelihood indicates improved agreement between the model and observed data, and a smaller -2logL value signifies superior model performance.

##### 2.8.2.2 Akaike information criterion

This model selection method relies on the connection between maximum likelihood estimation and Kullback–Leibler information. It is established with the premise that the operating models align with the approximating family (25, 26).


$$\mathrm{AIC}=-2\;\log\;L\left(\theta \right)+\mathrm{2K}$$

In this context, L(θ) represents the maximized likelihood function, and K denotes the count of estimated parameters in the model, encompassing variables and the intercept. The log likelihood of the data model serves as a measure of the model’s overall fit, where a lower log likelihood value signifies a less optimal model fit. However, upon comparing various models, the one exhibiting the minimum AIC value is considered the most favorable.

##### 2.8.2.3 Bayesian information criterion

BIC shares similarities with AIC, with the distinction lying in the second term, which is contingent on the sample size denoted as ‘n’.


BIC=−2log(L)+plog(n)

In this context, where ‘L’ represents the log likelihood, ‘p’ signifies the number of parameters, and ‘n’ denotes the number of observations utilized in the model, a smaller Bayesian Information Criterion (BIC) value indicates a superior model. The derivation of BIC assumes uniform priors on each model and non-informative priors on the parameters within each model (27). The primary objective of BIC is to identify the optimal predictive model by maximizing the posterior probability, whereas the goal of AIC is to pinpoint the model that most credibly explains the data generation process.

##### 2.8.2.4 Model comparison and justification

The negative binomial regression (NBR) model was selected over the Poisson model based on these criteria. Table 1 presents the results, showing that the Chi-square -2 log-likelihood statistic for the NBR model is substantially lower (2980.85) compared to the Poisson model (6025.85), indicating a better fit. Additionally, both the AIC and BIC values are lower for the NBR model (AIC = 6399.598, BIC = 6520.91) compared to the Poisson model (AIC = 6875.472, BIC = 6990.718), further supporting the selection of the NBR model.

**Table 1:** Model specification tests.

Model	Chi-square -2log likelihood statistic	AIC	BIC
Poison	6025.9	6875.5	6990.7
Negative binomial regression*	2980.9	6399.6	6520.9

*Model selected.

In assessing the suitability of statistical models for our analysis of ANC visits, dispersion statistics played a crucial role. Overdispersion, where the variance of the data exceeds its mean, was evident in our dataset, particularly in ANC visit patterns among women of varying wealth index categories. The dispersion parameter (*k*) estimated for ANC visits using the Poisson regression model was approximately 3.39, indicating substantial variability beyond what would be expected under a standard Poisson distribution. This level of overdispersion suggests that the Poisson model does not adequately capture the variance in the data. In contrast, the Negative Binomial regression model, which accounts for overdispersion, yielded a dispersion parameter (*α*) of 2.439, providing a better fit for the observed variability in ANC visit counts (28). Visual inspection of the histogram depicting ANC visit distribution revealed a right-skewed pattern, with a variance–mean ratio of 3.56, further confirming the presence of overdispersion. Vuong’s test supported these findings with a *p*-value <0.001, favoring the NBR model over the standard Poisson model due to its superior fit for overdispersed count data (29). These dispersion statistics provided robust evidence for selecting the NBR model, which accommodates varying levels of dispersion more effectively than the standard Poisson model in healthcare utilization studies. By explicitly modeling the variance, the NBR approach offers a realistic depiction of how ANC utilization varies across different wealth categories in our study population. All statistical analyses were conducted at a 5% significance level using Stata version 18.

#### 2.8.3 Model specification

##### 2.8.3.1 Negative binomial regression

The negative binomial regression model assumes a gamma distribution for *θ*_i_ introducing class-specific heterogeneity in contrast to the standard Poisson distribution’s homogeneous mean assumption within classes (24, 28). In this context, “class” refers to the categories or groups within which the data is assumed to be homogeneous or heterogeneous. Specifically, for the standard Poisson distribution, it assumes that within each class (within each set of observations sharing the same characteristics), the mean count is homogeneous (the same across all observations). However, in real-world data, count data often exhibit overdispersion, where the variance exceeds the mean. The negative binomial regression model accommodates this by introducing class-specific heterogeneity, allowing for varying levels of dispersion within each class. This flexibility accommodates varying levels of dispersion in count data, making it suitable for our analysis. Moreover, multilevel analysis was not chosen, because the ANC visits data lacks a hierarchical structure that would benefit from such an approach. Each individual’s ANC visits were treated independently, without hierarchical clustering that would justify multilevel modeling. Thus, the negative binomial regression model was chosen for its ability to handle overdispersion in count data effectively.


$$P\left(Y=y|X_{1},X_{2},X_{3},k\right)=\frac{\Gamma \left(y+k\right)}{\Gamma \left(k\right)\Gamma \left(y+1\right)}{\left(\frac{k}{k+\mu }\right)}^{k}{\left(\frac{\mu }{k+\mu }\right)}^{y}y=0,1,2,..$$

Definitions:

-
Y : The dependent variable representing the number of ANC visits, which is a count variable.
-
$X_{1},X_{2},X_{3},k$ : The covariates included in the model such as $X_{1}$ : Poverty status (wealth index), $X_{2}$ : Residence and $X_{3}$ : Education.
-
k : The dispersion parameter, which allows the variance to differ from the mean (accounting for over-dispersion in the count data).
- μ: The mean of the dependent variable Y , which is modeled as a function of the covariates:

μ = exp.(*β*0  + *β*1 $X_{1}$ + *β*2 $X_{2}$ + *β*3 $X_{3}$ ) where:

*β*0 : The intercept term.

*β*1, *β*2, *β*3: The coefficients for the covariates $X_{1},X_{2},X_{3}$ , respectively.

- Γ (·): The gamma function, which generalizes the factorial function to non-integer values.

The crude Incidence Rate Ratio (cIRR) were calculated without adjusting for any covariates. They provided a measure of the association between each individual covariate and the number of ANC visits, without inclusion of the potential confounding effects of other variables in the analysis. The aIRR were derived from the negative binomial regression model shown below, which included multiple covariates simultaneously. This adjustment accounted for the confounding effects, providing a more accurate measure of the association between each covariate and the number of ANC visits.

##### 2.8.3.2 Multinomial logit model

The multinomial logit model was employed to assess the nexus between of poverty and choice of place of birth, categorizing facilities as public, private, or home (30, 31). This model is appropriate for our study because it allows for the analysis of outcomes where the dependent variable is categorical and has more than two levels.

The probability that women n chooses delivery place j among j possible choices is given by:


$$P\left(Choiceofplaceofbirth=j\right)=\frac{\exp\left(\mathrm{Vjn}\right)}{\;\Sigma_{k=1}^{j}\exp\;\left(Vkn\right)}$$

Where Vjn is the utility derived from choosing delivery place j for Women n.

Utility function:

The utility derived by women n from choosing delivery place j is specified as:


$$P\left(Choiceofplaceofdelivery=j\right)=\exp\left(\beta_{0}+\Sigma_{i=1}^{11}\beta_{1}X_{i}+\epsilon \right)$$

Also, *P*(Choice of place delivery = *j*) represents the probability of choosing a specific place delivery (government, private, or home, where *j* refers to each category).

$\beta_{0}$ = Intercept term.

$\beta_{1}\;$ to $\beta_{11}$ = Coefficients estimated for covariates $X_{1}$ to $X_{11}$ _._

$X_{1}$ :Covariates representing factors influencing choice of place of birth.

Probability of choice:

The probability that individual n will choose delivery place j is:


$$P\left(Choiceofplaceof\;\mathrm{birth}=j\right)=\frac{\exp\left(\beta_{0}+\Sigma_{i=1}^{11}\beta_{1}X_{i}+\epsilon \right)}{\Sigma_{k=1}^{j}\exp\;\left(\exp\beta_{0}+\Sigma_{i=1}^{11}\beta_{1}X_{i}+\epsilon \right)}$$

Where:

$\beta_{0}$ = Intercept term.

$\beta_{1}\;$ to $\beta_{11}$ = Coefficients estimated for covariates $X_{1}$ to $X_{11.}$

$X_{1}:\mathrm{Covariates such}\;\mathrm{as}\;WI\;$ (Poverty status), *R* (Residence), *Ed* (Education level), *Reg* (Region), *A* (Age), *Tch* (Total children), *BO* (Birth order), *IU* (Internet use), *MO* (Mobile phone ownership), *NM* (Availability of nurse midwives), *CO* (Availability of clinical officers).

ϵ = Error term.

## 3 Results

### 3.1 Background characteristics of study participants

The background characteristics of the study participants in relation poverty is shown in Table 2. The mean maternal age is similar for both the women in the poorer and richer households, with an overall average of 29.7. However, differences were noted in antenatal care visits, with women from richer households reporting a higher average of 2.3 visits compared to 0.7 visits in the poorer households. Regarding the choice of place of birth, majority (86.5%) of participants in the poor households prefer home delivery, in contrast to the women in richer households where 46.1% opted for home delivery. Public health facilities were more commonly utilized by women in the richer households (39.8%) compared to women in poorer households (11.9%). A higher proportion of women with no education belong to the poorer household’s wealth quintile (91.9%). Most of the women living in urban areas were from richer households (61.5%), while nomadic living was predominant in the poorer households (58.5%). Access to technology followed a similar trend, with a high percentage (98.4%) of women in the poorer households reporting no internet use, while mobile phone ownership was higher among women in richer households (89.6%) compared to those in poorer households. Furthermore, the majority of mothers not employed were from poorer households (96.0%). Analysis of total children ever born showed a mean of 4.71 for the women in poorer households, and 4.33 for the women in richer households. Similarly, birth order showed a mean of 4.69 for the women in poorer households, and 4.34 for those richer households. Regarding ANC visits, the mean was approximately 1.27 visits per person. The standard deviation was 2.13, indicating variability around the mean. The minimum number of visits recorded was 0, while the maximum was 30, resulting in a range of 30 visits.

**Table 2:** Background characteristics of study participants.

Variables	Poorer	Richer	Total
	Wealth quintile	Wealth quintile	*n* = 3,183
	*n* = 2048, % = 100	*n* = 1,135, % = 100	% = 100
Maternal age (mean, standard deviation)	29.8 (7.0)	29.6 (6.5)	29.7 (6.8)
Antenatal care visits (mean, standard deviation)	0.7 (1.6)	2.3 (2.5)	1.3 (2.1)
Place of birth			
Home delivery	1771 (86.5%)	523 (46.1%)	2,294 (72.1%)
Public health facility	243 (11.9%)	452 (39.8%)	695 (21.8%)
Private health facility	34 (1.7%)	160 (14.1%)	194 (6.1%)
Total	2048 (100.0%)	1,135 (100.0%)	3,183 (100.0%)
Region			
Awdal	274 (13.4%)	130 (4.1%)	404 (12.7%)
MarodiJeh	224 (10.9%)	162 (14.3%)	386 (12.1%)
Sahil	221 (10.8%)	183 (16.1%)	404 (12.7%)
Togdheer	372 (18.2%)	166 (14.6%)	538 (16.9%)
Sool	539 (26.3%)	168 (14.8%)	707 (22.2%)
Sanaag	418 (20.4%)	326 (28.7%)	744 (23.4%)
Total	2048 (100.0%)	1,135 (100.0%)	3,183 (100.0%)
Level of education			
No Education	1883 (91.9%)	747 (65.8)	2,630 (82.6%)
Primary	155 (7.6%)	273 (24.1%)	428 (13.5%)
Secondary	8 (0.4%)	74 (6.5%)	82 (2.6%)
Higher	2 (0.1%)	41 (3.6%)	43 (1.4%)
Total	2048 (100.0%)	1,135 (100.0%)	3,183 (100.0%)
Total children ever born (mean, standard deviation)	4.71 (2.77)	4.33 (2.73)	4.57 (2.76)
Birth order (mean, standard deviation)	4.69 (2.71)	4.34 (2.72)	4.56 (2.72)
Place of residence			
Nomadic	1,198 (58.5%)	21 (1.9%)	1,219 (38.3%)
Rural	650 (31.7%)	416 (36.7%)	1,066 (33.5%)
Urban	200 (9.8%)	698 (61.5%)	898 (28.2%)
Total	2048 (100.0%)	1,135 (100.0%)	3,183 (100.0%)
Internet use			
No	2016 (98.4%)	934 (82.3%)	2,950 (92.7%)
Yes	32 (1.6%)	201 (17.7%)	233 (7.3%)
Total	2048 (100.0%)	1,135 (100.0%)	3,183 (100.0%)
Mobile phone ownership			
No	694 (33.9%)	118 (10.4%)	812 (25.5%)
Yes	1,354 (66.1%)	1,017 (89.6%)	2,371 (74.5%)
Total	2048 (100.0%)	1,135 (100.0%)	3,183 (100.0%)
Nurse midwife availability			
No	1,683 (82.2%)	621 (54.7%)	2,304 (72.4%)
Yes	365 (17.8%)	514 (45.3%)	879 (27.6%)
Total	2048 (100.0%)	1,135 (100.0%)	3,183 (100.0%)
Clinical officer availability			
No	2043 (99.8%)	1,128 (99.4%)	3,171 (99.6%)
Yes	5 (0.2%)	7 (0.6%)	12 (0.4%)
Total	2048 (100.0%)	1,135 (100.0%)	3,183 (100.0%)
Doctor availability			
No	1959 (95.7%)	951 (83.8%)	2,910 (91.4%)
Yes	89 (4.4%)	184 (16.2%)	273 (8.6%)
Total	2048 (100.0%)	1,135 (100.0%)	3,183 (100.0%)
Problems in accessing health care			
*Permission from husband to seek care*			
No	1,116 (54.5%)	741 (65.3%)	1,857 (58.3%)
Yes	932 (45.5%)	394 (34.7%)	1,326 (41.7%)
Total	2048 (100.0%)	1,135 (100.0%)	3,183 (100.0%)
*Getting money for treatment*			
No	507 (24.8%)	495 (43.6%)	1,002 (31.5%)
Yes	1,541 (75.2%)	640 (56.4%)	2,181 (68.5%)
Total	2048 (100.0%)	1,135 (100.0%)	3,183 (100.0%)
*Far Distance to health facility*			
No	518 (25.3%)	569 (50.1%)	1,087 (34.2%)
Yes	1,530 (74.7%)	566 (49.9%)	2,096 (65.9%)
Total	2048 (100.0%)	1,135 (100.0%)	3,183 (100.0%)
*Not wanting to go alone to health facility*			
No	1,003 (49.0%)	735 (64.8%)	1,738 (54.6%)
Yes	1,045 (51.0%)	400 (35.2%)	1,445 (45.4%)
Total	2048 (100.0%)	1,135 (100.0%)	3,183 (100.0%)
Mother employed			
No	1961 (96.0%)	1,035 (91.2%)	2,996 (94.1%)
Yes	87 (4.0%)	100 (8.8%)	187 (5.9%)
Total	2048 (100.0%)	1,135 (100.0%)	3,183 (100.0%)

**Figure 1:**
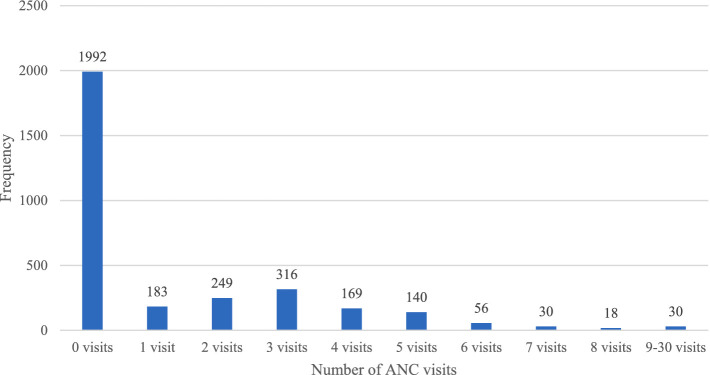
Distribution of antenatal visits during pregnancy.

The distribution of ANC visits is illustrated in Figure 1. The histogram reveals a prominent peak at 0 occurrences, indicating a significant number of instances (1,992) where no ANC visits were recorded. As the count increases, the frequency gradually decreases, illustrating a skewed distribution toward lower counts. This pattern shows that the majority of observations exhibit low ANC visit counts, with sporadic instances of higher counts dispersed throughout the data, which is characteristic of overdispersion.

### 3.2 Association between poverty and ANC visits in Somaliland

The analysis shown in Table 3 shows the crude and adjusted incidence rate ratio (aIRR) of factors influencing antenatal care (ANC) visits, with the overall aim of showing the effect of poverty proxied by wealth quintile on antenatal care utilization. Wealth quintile showed significant disparities, as women in the poorer households had 22% lower incidence rate of ANC visits (aIRR = 0.884, 95% CI: 0.791–0.987) compared to women in richer households. The maternal age did not show a significant impact on ANC visits (aIRR = 1.002, 95% CI: 0.993–1.011).

**Table 3:** Parameter estimates for negative binomial regression on the association between of poverty and ANC visits, Crude and Adjusted IRR.

Variables	Crude IRR, lower and upper 95% confidence interval	Adjusted IRR, lower and upper 95% confidence interval
Mothers age	0.996 (0.986–1.007)	1.00 (0.993–1.011)
Region		
Awdal	1.303 (1.009–1.683)*	1.078 (0.924–1.258)
Sahil	1.145 (0.886–1.481)	0.876 (0.749–1.020)
Togdheer	0.842 (0.660–1.074)	0.803 (0.687–0.939)**
Sool	0.420 (0.331–0.534)***	0.710 (0.601–0.839)***
Sanaag	0.480 (0.380–0.607)***	0.654 (0.558–0.768)***
Maroodi Jeex	1.00	1.00
Level of education		
No education	0.265 (0.155–0.453)***	0.840 (0.629–1.122)
Primary	0.568 (0.325–0.993)*	0.916 (0.689–1.218)
Secondary	0.780 (0.404–1.507)	0.924 (0.674–1.267)
Higher	1.00	1.00
Total children ever born	0.957 (0.933–0.981)**	0.963 (0.897–1.034)
Birth order	0.963 (0.939–0.987)**	1.021 (0.954–1.094)
Wealth quintile		
Poorer	0.302 (0.265–0.344)***	0.884 (0.791–0.987)*
Richer	1.00	1.00
Residence		
Nomadic	0.108 (0.092–0.128)***	0.454 (0.383–0.538)***
Rural	0.638 (0.556–0.732)***	0.975 (0.880–1.081)
Urban	1.00	1.00
Internet use		
Yes	2.559 (2.00–3.273)***	1.01 (0.860–1.181)
No	1.00	1.00
Mobile phone ownership		
Yes	1.932 (1.640–2.276)***	1.149 (1.013–1.302)**
No	1.00	1.00
Nurse midwife availability		
Yes	8.992 (8.155–9.914)***	13.821 (12.093–15.796)***
No	1.00	1.00
Clinical officer availability		
Yes	2.373 (0.817–6.893)	2.956 (1.668–5.236)***
No	1.00	1.00
Doctor availability		
Yes	3.029 (2.421–3.789)***	9.467 (8.051–11.133)***
No	1.00	1.00
Problems in accessing health care		
*Seeking permission from husband to seek care*		
Yes	0.706 (0.613–0.813)***	0.889 (0.787–1.005)
No	1.00	1.00
*Getting money for treatment*		
Yes	0.678 (0.585–0.785)***	1.086 (0.950–1.242)
No	1.00	1.00
*Far distance to health facility*		
Yes	0.584 (0.506–0.673)***	0.967 (0.844–1.108)
No	1.00	1.00
*Not wanting to go alone to health facility*		
Yes	0.613 (0.533–0.704)***	0.932 (0.826–1.051)
No	1.00	1.00
Mother employed		
Yes	1.661 (1.251–2.206)***	0.925 (0.786–1.088)
No	1.00	1.00

Reference *p < 0.05; **p < 0.01; ***p < 0.001.

Women in Togdheer, Sool and Sanaag regions exhibited lower incidence rate of antenatal care visits by 20, 29, and 35%, respectively, (aIRR = 0.803, 95% CI: 0.687–0.939, aIRR = 0.710, 95% CI: 0.601–0.839, and aIRR = 0.654, 95% CI: 0.558–0.768 respectively), compared to Maroodi Jeex region. Regarding the level of education, women with no education and primary education displayed non-significant lower incidence rate in ANC visits (aIRR = 0.840, 95% CI: 0.629–1.122, and aIRR = 0.916, 95% CI: 0.689–1.218, respectively). Similarly, women with secondary education exhibited a non-significant lower incidence rate in ANC visits (aIRR = 0.924, 95% CI: 0.674–1.267). The total number of children ever born did not significantly impact ANC visits (aIRR = 0.963, 95% CI: 0.897–1.034). However, birth order emerged as a significant factor, with women having a second or subsequent birth order having 21% lower incidence rate of ANC visits compared to those with a first birth (aIRR = 0.885, 95% CI: 0.804–0.973).

Examining residence, nomadic women had 55% lower incidence rate of attending ANC visits compared to urban residents (aIRR = 0.454, 95% CI: 0.383–0.538), while women residing in rural areas did not exhibit a significant difference compared to urban residents (aIRR = 0.975, 95% CI: 0.880–1.081). Women with internet access did not show a significant difference in ANC visits compared to those without internet access (aIRR = 1.008, 95% CI: 0.860–1.181). Mobile phone ownership, however, emerged as a significant factor, with women having mobile phones exhibiting 85% higher incidence rate of ANC visits (aIRR = 1.149, 95% CI: 1.013–1.302) compared to those without mobile phones. The availability of healthcare providers significantly influenced ANC visits, with women attended by nurse midwives (aIRR = 13.821, 95% CI: 12.093–15.796) showing an 18% higher incidence rate, clinical officers (aIRR = 2.956, 95% CI: 1.668–5.236) demonstrating a 5% higher incidence rate, and doctors (aIRR = 9.467, 95% CI: 8.051–11.133) exhibiting a 53% higher incidence rate in ANC visits.

Education, total children, birth order, Internet use, seeking permission from husband, getting money, far distance to health facility, not wanting to go alone, mothers employment were not statistically associated with ANC visits.

### 3.3 Association between poverty and choice of place of birth in Somaliland

Table 4 highlights the crude and adjusted relative risk ratios for factors influencing the choice of place of birth. The wealth quintile demonstrated significant disparities: women from poorer households had 54% lower relative probability (aRRR: 0.457, 95% CI: 0.352–0.593) to opt for public health facilities over home births and 80% lower relative probability (aRRR: 0.195, 95% CI: 0.111–0.341) to choose private facilities over home births, compared to women from richer households.

**Table 4:** Parameter estimates of a multinomial logistics regression on the association between poverty and choice of place of birth, Crude and Adjusted RRR.

	Public health facility		Private health facility	
	Crude RRR()	Adjusted RRR	Crude RRR	Adjusted RRR
Mothers age	0.980 (0.968–0.993)**	0.984 (0.963–1.006)	0.995 (0.973–1.016)	1.029 (0.993–1.067)
Region				
Awdal	1.460 (1.069–1.991)	1.301 (0.886–1.910)	0.403(0.244–0.664)***	0.350 (0.191–0.642)
Sahil	1.595 (1.172–2.171)	1.158 (0.796–1.686)	0.261 (0.146–0.467)***	0.202 (0.104–0.392)
Togdheer	0.511 (0.370–0.705)	0.331 (0.223–0.491)	0.333 (0.214–0.516)***	0.234 (0.136–0.405)
Sool	0.346 (0.251–0.476)	0.356 (0.242–0.521)	0.168 (0.104–0.271)***	0.182 (0.103–0.321)
Sanaag	0.287 (0.207–0.397)	0.175 (0.118–0.259)	0.161 (0.100–0.259)***	0.084 (0.048–0.150)
Maroodi Jeex	1.00	1.00	1.00	1.00
Level of education				
No education	0.056 (0.021–0.148)***	0.255 (0.076–0.857)	0.016 (0.006–0.043)***	0.139 (0.037–0.512)
Primary	0.160 (0.059–0.433)***	0.336 (0.100–1.126)	0.065 (0.023–0.185)***	0.260 (0.072–0.939)
Secondary	0.524 (0.173–1.588)	0.770 (0.208–2.854)	0.265 (0.081–0.864)*	0.457 (0.111–1.883)
Higher	1	1.00	1.00	1.00
Total children ever born	0.907 (0.878–0.937)***	1.020 (0.835–1.245)	0.878 (0.827–0.932)***	0.951 (0.690–1.311)
Birth order	0.907 (0.877–0.938)***	0.916 (0.752–1.117)	0.879 (0.828–0.933)***	0.930 (0.675–1.272)
Wealth quintile				
Poorer	0.159 (0.132–0.191)***	0.457 (0.352–0.593)	0.063 (0.043–0.092)***	0.195 (0.111–0.341)
Richer		1.00		1.00
Residence				
Nomadic	0.055 (0.041–0.073)	0.159 (0.109–0.232)	0.044 (0.027–0.073)***	0.350 (0.171–0.717)
Rural	0.342 (0.280–0.418)	0.439 (0.341–0.565)	0.110 (0.074–0.165)	0.203 (0.128–0.322)
Urban	1.00	1.00	1.00	1.00
Internet use				
Yes	4.553 (3.333–6.221)***	0.865 (0.556–1.350)	12.416 (8.489–18.160)***	1.270 (0.728–2.214)
No	1.00	1.00	1.00	1.00
Mobile phone ownership				
Yes	2.170 (1.741–2.705)***	1.303 (0.984–1.725)	3.881 (2.397–6.283)	1.559 (0.896–2.714)
No	1.00	1.00	1.00	1.00
Nurse midwife availability				
Yes	6.274 (5.214–7.549)***	3.450 (2.746–4.332)	4.063 (3.002–5.497)	2.508 (1.704–3.694)
No	1.00	1.00	1.00	1.00
Clinical officer availability				
Yes	5.825 (1.700–19.957)**	4.146 (0.848–20.277)	2.966 (0.330–26.670)	1.931 (0.156–23.870)
No	1.00	1.00	1.00	1.00
Doctor availability				
Yes	3.602 (2.713–4.783)***	3.045 (2.142–4.330)	9.063 (6.319–12.997)	5.030 (3.150–8.028)
No	1.00	1.00	1.00	1.00
Problems in accessing health care				
*Seeking permission from husband to seek care*				
Yes	0.859 (0.723–1.021)	1.199 (0.898–1.601)	0.450 (0.323–0.627)	0.553 (0.331–0.923)
No	1.00	1.00	1.00	1.00
*Getting money for treatment*				
Yes	0.653 (0.546–0.781)***	1.135 (0.813–1.586)	0.382 (0.284–0.514)	1.207 (0.718–2.028)
No	1.00	1.00	1.00	1.00
*Far distance to health facility*				
Yes	0.539 (0.453–0.642)	0.854 (0.610–1.194)	0.325 (0.241–0.438)	0.882 (0.517–1.504)
No	1.00	1.00	1.00	1.00
*Not wanting to go Alone to health facility*				
Yes	0.673 (0.566–0.801)***	1.067 (0.799–1.425)	0.438 (0.318–0.603)***	1.102 (0.678–1.791)
No	1.00	1.00	1.00	1.00
Mother employed				
Yes	1.757 (1.261–2.447)**	0.943 (0.624–1.423)	2.135 (1.282–3.557)**	1.059 (0.562–1.992)
No	1.00	1.00	1.00	1.00

Reference *p < 0.05; **p < 0.01; ***p < 0.001.

Women in the regions of Togdheer, Sool, and Sanaag had lower probabilities of choosing public health facilities for delivery over home births, with probabilities decreased by 67, 76, and 82%, respectively (aRRR 0.331, 95% CI 0.223–0.491; aRRR 0.356, 95% CI 0.242–0.521; aRRR 0.175, 95% CI 0.118–0.259), compared to women in the Maroodi Jeex region. Similarly, women in the regions of Awdal, Sahil, Togdheer, Sool, and Sanaag had lower probabilities of choosing private health facilities for delivery over home births, with probabilities decreased by 65, 80, 77, 86, and 92%, respectively (aRRR 0.350, 95% CI 0.191–0.642; aRRR 0.202, 95% CI 0.104–0.392; aRRR 0.234, 95% CI 0.136–0.405; aRRR 0.182, 95% CI 0.103–0.321; aRRR 0.084, 95% CI 0.048–0.150), compared to women in the Maroodi Jeex region.

Women with no education had a 74% lower probability of choosing public health facilities for delivery over home births (aRRR 0.255, 95% CI: 0.076–0.857) compared to women with higher education. Similarly, women with no education and those with primary education had lower probabilities of choosing private health facilities for delivery over home births, with decreased probabilities to 86% (aRRR 0.139, 95% CI: 0.037–0.512) and 74% (aRRR 0.260, 95% CI: 0.072–0.939), respectively in comparison to women with higher education.

Residence significantly influenced the choice of place of birth compared to home births, when comparing to urban settings. Regarding public health facilities, nomadic residents exhibited a substantial 84% lower probability (aRRR 0.159, 95% CI: 0.109–0.232) of choosing public health facilities for delivery over home births compared to urban residents. Similarly, rural residents had a 56% lower probability (aRRR 0.439, 95% CI: 0.341–0.565) of opting for public health facilities over home births compared to women in urban areas. In terms of private health facilities, this trend continued with nomadic residents showing a 65% lower probability (aRRR 0.350, 95% CI: 0.171–0.717) of choosing private health facilities over home births compared to urban residents. Likewise, rural residents had a 79% lower probability (aRRR 0.203, 95% CI: 0.128–0.322) of choosing private health facilities over home births compared to their urban counterparts.

Women who sought care from nurse/midwives had 65 and 49% higher probabilities of choosing public health facilities and private health facilities, respectively, for delivery over home births (aRRR 3.450, CI:95% 2.746–4.332, and aRRR 2.508 CI: 95% 1.704–3.694 respectively). The type of healthcare provider significantly influenced women’s choices regarding place of birth. Women receiving care from doctors had 95 and 97% higher probabilities of opting for both public and private health facilities, respectively, compared to those not receiving care from doctors (aRRR 3.045, 95% CI: 2.142–4.330 and aRRR 5.030, 95% CI: 3.150–8.028 respectively). Regarding private health facilities, women who required permission from their husbands to visit a health facility had a 44.7% lower probability (95% CI: 33.1–92.3%) of choosing private health facilities for delivery over home births compared to women who did not need such permission. Total children ever born, birth order, internet use, getting money, clinical officer availability, not wanting to go alone to the health facility and mothers’ employment were not associated with the choice of public or private health facility over home births.

## 4 Discussion

To the best of our knowledge, this study is the first to investigate the relationship between poverty and maternal healthcare utilization, specifically focusing on ANC visits and the choice of place of birth in Somaliland. Our findings provide important evidence on the importance of addressing wealth inequality to increase ANC visits and encourage women to choose institutional deliveries over home births. In Somaliland, wealth quintile disparities were evident, with women from poorer households experiencing a lower incidence rate of ANC visits compared to women from richer households, similar to findings from previous studies (32–34). Additionally, women from poorer households had a decreased probability of opting for public and private health facilities, respectively, for birth compared to those from richer households. In Somaliland, the effect of poverty on ANC visits and the choice of birthplace was expected to be insignificant, considering the free maternal health policy aimed to encourage women to utilize maternal healthcare services. However, our findings suggest that poverty still has a negative and significant influence on the utilization of ANC visits and the choice of birthplace, such as public or private health facilities, contrary to our initial expectations. This indicates that despite the services being offered for free, there may be additional costs that those from poorer households are unable to afford. Women can still bear costs for medications, laboratory tests, and supplies during ANC visits and childbirth, which can be prohibitive for women from poorer households who lack sufficient financial resources (7).

To improve ANC visits, it is necessary to go beyond providing free services by finding additional support mechanisms for women. This could include providing necessary drugs and ensuring their availability at health facilities, thus reducing costs associated with purchasing drugs when they are not available. Financial constraints on maternal healthcare access are a widespread issue globally. Studies, such as one conducted by Kiruja et al. (7), highlight that in contexts such as Somaliland, women often prefer home-based care to avoid expenses incurred at healthcare facilities. Studies underscore the impact of user fees on maternal healthcare utilization among vulnerable populations. For instance, studies in sub-Saharan Africa have documented how user fees for maternal health services deter women, especially those from low-income households, from seeking timely and appropriate care (35–37). These financial barriers contribute to increased maternal and neonatal mortality rates and perpetuate inequalities in healthcare access (38). Efforts to address these challenges require comprehensive strategies. Implementing policies to eliminate user fees for maternal health services, as demonstrated in Ghana’s experience, can significantly improve access and utilization among disadvantaged populations (39). Additionally, promoting community-based health insurance schemes and strengthening health system capacity in underserved areas are essential steps toward achieving equitable maternal healthcare access (40, 41). By addressing financial barriers and enhancing healthcare affordability, countries can make substantial strides toward improving maternal health outcomes and achieving sustainable development goals.

Women in the regions of Togdheer, Sool, and Sanaag exhibited lower probabilities of choosing public health facilities for delivery over home births, with significant decreases compared to women in the Maroodi Jeex region. This finding is in line with findings from previous studies showing regional differences in choice of place of birth (42, 43). These regional disparities were also evident in ANC visits, where women in these regions had significantly fewer visits compared to those in Maroodi Jeex, similar to previous studies that have shown regional disparities in ANC visits (44, 45). This suggests that women in Togdheer, Sool, and Sanaag face greater barriers in accessing maternal health services, whether for ANC or delivery, likely due to differences in healthcare infrastructure, availability of services, and socio-economic conditions. Similarly, women in Awdal and Sahil also showed lower probabilities of choosing private health facilities for delivery over home births, reinforcing the notion of regional disparities in healthcare access (42, 43).

Educational attainment significantly influenced the choice of place of birth, with women having no education showing a much lower probability of choosing public health facilities for delivery over home births compared to women with higher education. This trend was consistent for private health facilities as well, where women with no education and those with primary education had lower probabilities of opting for private health facilities for delivery (46, 47). This consistent trend underscores the pivotal role of educational attainment in shaping maternal healthcare decisions across different healthcare settings. Moreover, higher educational levels have been linked to increased awareness of maternal health risks, better understanding of healthcare options, and improved access to healthcare information, all of which contribute to more informed choices to public and private healthcare facilities as places of delivery.

Residence significantly influenced both ANC visits and the choice of place of birth. Nomadic women had a substantially lower probability of attending ANC visits and choosing public or private health facilities for delivery over home births compared to urban residents. Rural residents also showed lower probabilities of choosing public or private health facilities for delivery, though their ANC visit rates were not significantly different from urban women. This highlights the substantial barriers faced by nomadic populations in accessing consistent healthcare services, whether for ANC or delivery, and underscores the primary divide between nomadic and settled populations rather than between rural and urban residents (48).

The type of healthcare provider emerged as a crucial factor in determining both ANC visits and the choice of place of birth. Women receiving care from nurse/midwives or doctors had significantly higher probabilities of choosing public and private health facilities for delivery over home births, underscoring the importance of skilled healthcare providers in encouraging regular and institutional maternal healthcare utilization (49). This finding highlights the critical role of skilled healthcare providers in promoting consistent and institutional maternal healthcare utilization. Moreover, such providers are often better equipped to provide comprehensive prenatal care, offer guidance on delivery options, and address potential complications, thereby contributing to improved maternal and neonatal outcomes.

The study identified several factors that were not statistically associated with ANC visits and the choice of place of birth among women in Somaliland. Education level, total children ever born, birth order, internet use, seeking permission from husbands, financial constraints such as getting money, distance to health facilities, reluctance to go alone, and maternal employment did not show significant associations with ANC visits or the preference for public or private health facilities over home births. For ANC visits, education level, which often influences healthcare-seeking behaviors, did not demonstrate a significant impact in this study. This could be due to other underlying socio-economic factors such as poverty, healthcare accessibility, and cultural norms affecting maternal healthcare utilization in Somaliland (50). Similarly, factors like total children ever born, birth order, seeking permission from husband, which typically affect maternal healthcare utilization in other contexts (51–53), did not show significant associations. This suggests that while these factors may influence ANC visits elsewhere, they do not play a substantial role in Somaliland, possibly due to unique socio-cultural dynamics. Moreover, regarding the choice of place of birth, factors such as seeking permission from husbands and maternal employment did not emerge as significant determinants. This contrasts with findings from other settings where patriarchal norms and employment status may heavily influence women’s healthcare choices of place of birth (51, 54). The lack of association in Somaliland could reflect evolving gender dynamics or other decision-making processes within households that were not investigated in this study Future research should explore these non-statistically significant factors within Somaliland’s socio-cultural context to better understand their influence on maternal healthcare decisions

### Strengths and limitations

This study used the most recent demographic health survey (SLDHS 2020), representing all regions of Somaliland, providing nationally relevant findings. The use of negative binomial and multinomial regression models strengthened the analysis, ensuring robust conclusions. However, the reliance on variables provided by the SLDHS may have excluded other relevant factors. Despite this limitation, the study offers crucial insights for policymakers and can be applicable to settings with similar sociodemographic characteristics to Somaliland.

## 5 Conclusion

In conclusion, our study shows the effect of poverty on maternal healthcare utilization, specifically regarding ANC visits and choice of place of birth. Despite initiatives to provide free maternal health services, poverty continue to impede access. Women from poorer households exhibit lower rates of ANC visits and are less likely to choose institutional deliveries over home births, underscoring significant financial barriers that deter healthcare utilization among vulnerable populations. Regional disparities exacerbate these challenges, with women in Togdheer, Sool, and Sanaag regions demonstrating reduced probabilities of opting for public and private health facilities compared to other regions. Efforts to enhance maternal healthcare in Somaliland should prioritize eliminating user fees for maternal health services and improving the healthcare system. These comprehensive strategies not only address financial barriers but also support sustainable improvements in maternal healthcare access. By focusing on these interventions, policymakers and healthcare providers can strive toward achieving equitable maternal health outcomes and reducing disparities across Somaliland.

## Data Availability

The data analyzed in this study is subject to the following licenses/restrictions: Data is available upon request. Requests to access these datasets should be directed to Ministry of National Planning and Development, www.slministryofplanning.org.
